# Correction: A colorimetric and far-red fluorescent probe for the highly sensitive detection of silver(i)

**DOI:** 10.1039/c8ra90002g

**Published:** 2018-01-16

**Authors:** Yong-jun Wang, Jing-gong Liu, Hui-ya Tan, Jin-wu Yan, Lei Zhang

**Affiliations:** School of Biology and Biological Engineering, South China University of Technology Guangzhou P. R. China yjw@scut.edu.cn +86 20 39380678; The Second Affiliated Hospital of Guangzhou University of Chinese Medicine, Guangdong Provincial Academy of Chinese Medical Sciences Guangzhou 510006 P. R. China

## Abstract

Correction for ‘A colorimetric and far-red fluorescent probe for the highly sensitive detection of silver(i)’ by Yong-jun Wang *et al.*, *RSC Adv.*, 2017, **7**, 55567–55570.

The published article incorrectly indicates four corresponding authors. The two corresponding authors should be Jin-wu Yan and Lei Zhang. In addition, a link to the equal contribution footnote, confirming that Yong-jun Wang and Jing-gong Liu contributed equally to the work, is missing in the published article. These errors are corrected herein.

The Royal Society of Chemistry apologises for these errors and any consequent inconvenience to authors and readers.

## Supplementary Material

